# Co-Existent Central and Peripheral Demyelination: Related or Coincidental?

**DOI:** 10.3390/neurolint16060121

**Published:** 2024-12-03

**Authors:** Camila Narvaez-Caicedo, Shireen M. Jacob, Laura Wu, Chilvana Patel

**Affiliations:** Department of Neurology, University of Texas Medical Branch, Galveston, TX 77555, USA

**Keywords:** demyelinating disorders, sensory-motor demyelinating polyneuropathy, European Academy of Neurology and Peripheral Nervous System criteria for CIDP, response to IVIg, hereditary sensory-motor neuropathy type 1, multiple sclerosis

## Abstract

Background: Hereditary Sensory Motor Neuropathy (HSMN) 1A and Multiple Sclerosis (MS) are distinct demyelinating disorders affecting the peripheral and central nervous systems, respectively. We present a case of simultaneous occurrence of both conditions, exploring the clinical presentation, diagnostic workup, and potential interplay between these diseases. Case presentation and clinical approach: A 49-year-old male with a history of optic neuritis presented with progressive numbness, weakness, and sensory loss in all extremities over four years. Neurological examination revealed distal weakness, sensory deficits in a stocking-glove distribution, pes cavus, and hammer toes. Nerve conduction studies and electromyography confirmed sensory motor demyelinating polyneuropathy. The patient’s lack of response to intravenous immunoglobulin therapy suggested hereditary neuropathy as an etiology. Genetic testing identified a PMP22 gene duplication, confirming HSMN 1A. Elevated cerebrospinal fluid protein level and oligoclonal bands, combined with magnetic resonance of the brain showing multiple T2 hyperintense lesions in the brain and spinal cord, fulfilled the diagnostic criteria for MS. Discussion: This case of co-existing HSMN 1A and MS highlights a rare overlap of peripheral and central demyelination. While HSMN 1A results from PMP22 gene duplication, primarily affecting peripheral myelin, MS is driven by immune-mediated central myelin attacks. The co-existence of these disorders suggests potential shared mechanisms, such as immune dysregulation. Some evidence suggests that overexpression of PMP22 in HSMN 1A may disturb immune tolerance, possibly triggering autoimmune responses linked to MS. Further research is needed to explore the genetic and autoimmune interplay between these two diseases, expanding our understanding of demyelinating disorders.

## 1. Introduction

Hereditary Sensory Motor Neuropathy (HSMN) 1A, also known as Charcot–Marie–Tooth disease type 1A (CMT1A), is the most common inherited peripheral neuropathy, characterized by progressive distal weakness, sensory loss, and foot deformities. Multiple sclerosis (MS) is a chronic inflammatory demyelinating disease of the central nervous system, known for its multifocal lesions and clinical episodes of neurological dysfunction. While both diseases involve demyelination, their simultaneous occurrence is exceptionally rare, with limited reports in the literature. The co-existence of HSMN 1A and MS raises important questions about potential shared mechanisms, particularly in the context of immune responses and genetic predispositions. This case highlights a rare combination of these two disorders, offering insights into diagnostic challenges and the possible interplay between peripheral and central nervous system demyelination.

## 2. Case Presentation

A 49-year-old man presented with a four-year history of progressively lower and upper extremity numbness and weakness. Initially, the numbness manifested distally in the lower limbs and gradually progressed to knee level over the last year. He also complained of feet weakness causing gait impairment. Over the last six months, the numbness progressed to the mid-forearms, accompanied by weakness of hand grip and burning pain in all extremities. Other than a previously treated vitamin B12 deficiency and a resolved episode of left optic neuritis 12 years earlier, he was healthy and lacked familial neurological diseases.

His neurological examination revealed more distal than proximal weakness (Medical Research Council grades) in the upper and lower extremities: palmer and dorsal interosseous (4−), ankle flexion (4−), and ankle extension (4−), with the remaining muscles (5−). Proprioception was impaired in bilateral great toes, ankles, and index fingers, and temperature and pinprick sensations were decreased in a “stocking-glove” distribution. Deep tendon reflexes were decreased (1+) throughout the extremities; ankle reflexes were absent and plantar responses were flexor bilaterally. He had a high-steppage gait and Romberg’s sign was positive. Bilateral foot inspection revealed hammertoes and pes cavus.

## 3. Clinical Approach

The patient presented with chronic, progressive weakness and sensory loss in both the lower and upper extremities. The neurological exam showed distal more than proximal weakness, impaired sensation in a glove-stocking pattern affecting small and large fiber sensory modalities, and decreased deep tendon reflexes, suggestive of peripheral nervous system involvement. These features support a diagnosis of polyneuropathy, typically characterized by symmetric distal sensory loss, burning sensation, and/or weakness, as seen in this case.

Polyneuropathy has a wide range of causes, both acquired and inherited, categorized based on disease duration (acute or chronic) and underlying mechanism of injury (axonal or demyelinating). Differentiating these features informs the treatment approach and prognosis of the disease.

Distinguishing these features guides the treatment and prognosis of the disease. As the duration and progression of symptoms exceeded 8 weeks, a workup for chronic neuropathies was warranted. Nerve conduction studies (NCS) and electromyography (EMG) are important tools in differentiating axonal from demyelinating neuropathies and will prompt further diagnostic tests. Table 1 and Table 2 display the patient’s NCS/EMG results, which revealed severe sensory motor demyelinating neuropathy with significantly prolonged latency and slower conduction velocity, minimal temporal dispersion, and absence of conduction block. There was no active denervation in the examined muscles.

Laboratory tests showed normal renal and thyroid function, vitamin B12 levels, and serum and urine protein electrophoresis levels. Syphilis, hepatitis C virus, hepatitis B virus, and human immunodeficiency virus (HIV) serologies were negative. GM-1/anti-MAG antibodies were also negative. The patient denied a history of alcohol abuse, known exposure to heavy metals, or use of chronic medications except for multivitamins. Cerebrospinal fluid (CSF) analysis revealed one white blood cell, elevated protein level (69 mg/dL), elevated IgG index ratio (0.73, normal: 0.28–0.66), and seven oligoclonal bands.

**Figure 1:**
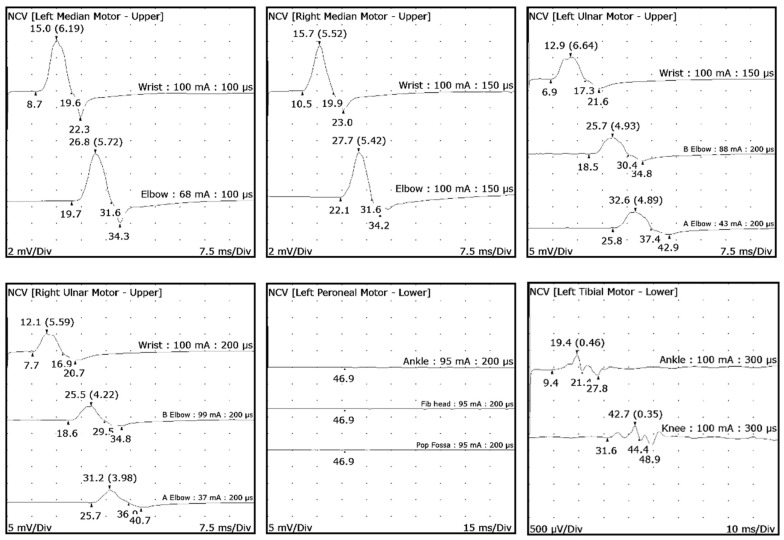
Nerve conduction study waveforms.

According to the European Academy of Neurology/Peripheral Nerve Society (EAN/PNS) criteria for chronic inflammatory demyelinating polyneuropathy (CIDP), the patient met the clinical criteria as he had progressive, symmetric, proximal, and distal weakness in upper and lower extremities with sensory involvement of at least two limbs, development over at least 8 weeks and decreased deep tendon reflexes in all extremities [1]. However, the electromyographic criteria necessary for CIDP diagnosis were not fulfilled due to minimal temporal dispersion and no conduction block (Table 1 and Table 2 and Figure 1). Additionally, the presence of hammer toes and pes cavus suggested a long-standing polyneuropathy, typically seen in hereditary polyneuropathies. Genetic testing detected a duplication of the PMP22 gene on chromosome 17, confirming the diagnosis of Hereditary Sensory Motor Neuropathy (HSMN) 1A. Other abnormal findings in the NCS/EMG study included decreased amplitude distally and chronic reinnervation indicating axonal involvement also. These findings were attributed to the patient’s previous history of vitamin B12 deficiency, considering the absence of abnormalities in the current laboratory studies.

**Figure 2:**
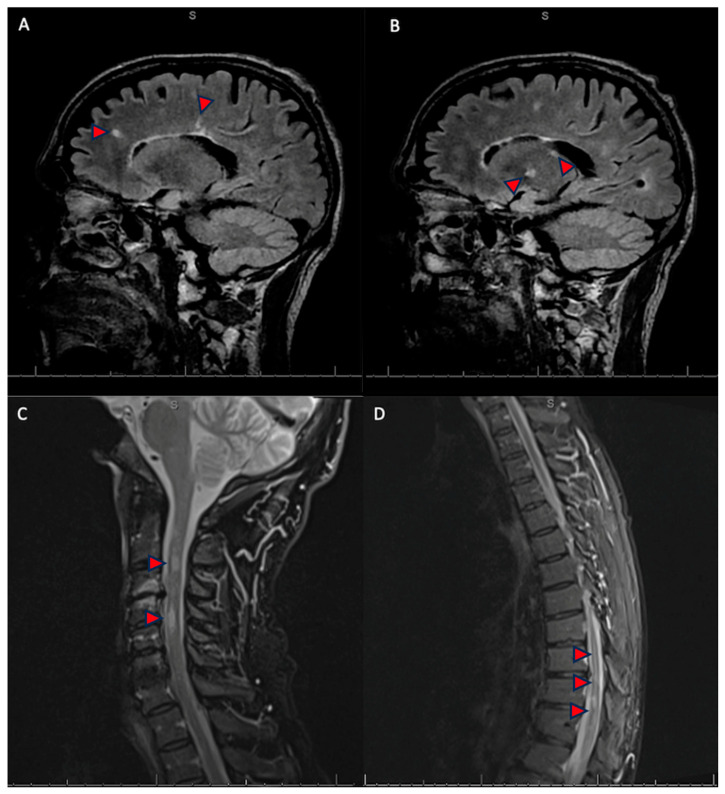
Sagittal brain MRI T2FLAIR sequence (**A**,**B**), and cervical (**C**) and thoracolumbar (**D**) MRI STIR T2 sequence. Multiple T2/FLAIR hyperintense lesions are noted at the subcortical and deep white matter of the bilateral cerebral hemispheres (**A**), and corpus callosum as well as in the left anterior thalamus (**B**). Three patchy short-segment T2 hyperintense lesions within the cervical spinal cord at C2-C3 and C4-C5 levels (**C**). There is diffuse volume loss of the thoracic cord with multifocal hyperintense lesions with the largest clusters at T10-T12 and extending into the conus medullaris (**D**). There are no enhancing lesions (**A**–**D**). The red arrows are indicate the abnormal findings described.

The presence of oligoclonal bands in CSF raised concerns about a simultaneous demyelinating disease in the central nervous system, particularly considering the patient’s previous episode of left optic neuritis, warranting imaging studies. Magnetic resonance imaging of the neuroaxis revealed multiple non-enhancing supra and infratentorial T2/FLAIR lesions and spinal T2/STIR hyperintense lesions (Figure 2). Based on the 2017 McDonald Criteria, these imaging findings, alongside the previous clinically isolated syndrome and presence of oligoclonal bands on CSF, led to a diagnosis of multiple sclerosis [2].

On presentation, based on the patient’s symptoms history, physical examination, and NCS/EMG results, the differential diagnoses included CIDP versus inherited polyneuropathy, leading to empiric initiation of intravenous immunoglobulin (IVIg) for CIDP while awaiting genetic test results. Unfortunately, there was no clinical improvement after receiving IVIg. According to EANPNS criteria, diagnosing CIDP requires meeting both clinical and electrodiagnostic criteria; however, in instances where one of these criteria is not entirely fulfilled, the diagnosis can be established based on the response to IVIg [1]. The lack of response to IVIg, the atypical confounding features seen in the NCS/EMG with a history of axonal neuropathy, and the examination findings hinting at a more longstanding neuropathy, prompted further investigation of inherited polyneuropathy, ultimately confirmed to be HSMN 1A.

To date, there is no cure for HSMN 1A, and treatment is focused on symptom management and complication prevention. Currently, our patient takes gabapentin for neuropathic pain, participates in physical therapy, and has a daily foot inspection for wound prevention.

Lastly, his newly diagnosed MS requires treatment. Disease-modifying therapy with Glatiramer commenced after a discussion with the patient, omitting steroid therapy due to the absence of acute neurological deficits or gadolinium-enhancing lesions on imaging studies.

## 4. Discussion

The potential link between multiple sclerosis (MS) and various subtypes of Charcot–Marie–Tooth disease (CMT) has been a subject of interest, particularly in the context of X-linked CMT (CMTX) [3]. Previous studies by Koutsis et al. [3] have explored this connection, describing five cases of CMTX patients who developed central nervous system (CNS) demyelination compatible with MS. The shared expression of connexin-32 (Cx32) in both the CNS and peripheral nervous system (PNS) led to the hypothesis that the GBJ1 mutation responsible for CMTX might serve as a risk factor for MS [3]. This intriguing proposition suggests an immune-mediated response triggered by inherited peripheral demyelination, potentially influencing CNS myelin [3].

However, the relationship between other CMT subtypes and MS remains less evident, with limited evidence supporting a direct association. We found fewer than a dozen reported cases like our patient with CMT 1A and MS. A study proposed that the overexpression of PMP22, common to both peripheral and CNS proteins, could modify self-tolerance, contributing to MS development [4]. Supporting this theory, there are additional cases reported of co-existing MS and CMT 1A, reinforcing the idea of an autoimmune response against CNS myelin stemming from the genetic defect of PMP22 [5,6].

Our presented case further contributes to this intriguing narrative, presenting both central and peripheral demyelinating features that meet the criteria for two distinct and, until now, unproven-to-be-related demyelinating disorders. The rarity of such co-existence is compounded by the atypical presentation in our patient, who developed late-onset CMT 1A symptoms without a family history and was diagnosed with MS without experiencing acute exacerbation. These unique aspects not only highlight the complexity of the possible interplay between genetic predispositions and autoimmune responses in neurological disorders, but also prompt a deeper investigation into the potential shared mechanisms underlying the co-existence of CMT subtypes and MS.

Future research should focus on elucidating these mechanisms through several avenues. Genetic studies could explore shared mutations or variants in genes such as PMP22 and BGJ1 to determine whether certain mutations predispose individuals to both conditions. Immunological investigations could shed light on shared inflammatory or autoimmune pathways, particularly those involving self-tolerance disruption in the presence of overlapping myelin proteins. Additionally, longitudinal cohort studies could assess whether individuals with inherited demyelinating conditions are at increased risk for developing central demyelination over time.

Understanding these shared mechanisms has the potential to enhance diagnostic precision, enable earlier identification of at-risk individuals, and pave the way for targeted therapies. These efforts could ultimately transform our approach to managing the broader spectrum of demyelinating disorders.

## Figures and Tables

**Table 1:** Nerve conduction studies. Abbreviations: nl (normal), amp (amplitude), neg (negative), dist (distance), vel (velocity), L (left), R (right), B (below), Fib head (fibular), and Pop (Popliteal).

Nerve/Sites		Onset (ms) [nl]	O-P Amp (mV) [nl]	iAmp (mV)	Neg Dur (ms)	Neg Area (mVms)	Site 1	Site 2	Delta-0 (ms)	Dist (cm)	Vel (m/s) [nl]
L Median Motor (Abd Poll Brev) 32.5 C	Wrist	8.7 [<4.0]	6.2 [>6.0]	7.6	10.9	30.92	Elbow	Wrist	11	26	24 [>48]
	Elbow	19.7 [<4.0]	5.7 [>6.0]	7	11.95	30.65					
R Median Motor (Abd Poll Brev) 35.5 C	Wrist	10.5 [<4.0]	5.5 [>6.0]	6.5	9.38	23.34	Elbow	Wrist	11	27.5	24 [>48]
	Elbow	22.1 [<4.0]	5.4 [>6.0]	6.3	9.49	25					
L Ulnar Motor (Abd Dig Min) 32.8 C	Wrist	6.9 [<3.6]	6.6 [>6.0]	7.8	10.43	39.66	Below Elbow	Wrist	11.6	25	22 [>51]
	Below Elbow	18.5 [<3.6]	4.9 [>6.0]	5.8	11.84	30.19	Above Elbow	Below Elbow	7.3	11	15 [>51]
	Above Elbow	25.8 [<3.6]	4.9 [>6.0]	5.7	11.6	31.39					
R Ulnar Motor (Abd Dig Min) 33.7 C	Wrist	7.7 [<3.6]	5.6 [>6.0]	6.4	9.14	29.25	Below Elbow	Wrist	10.9	23	21 [>51]
	Below Elbow	18.6 [<3.6]	4.1 [>6.0]	5	10.9	22.42	Above Elbow	Below Elbow	7.1	13	18 [>51]
	Above Elbow	35.7 [<3.6]	4 [>6.0]	4.5	11.25	19.98					
L Peroneal Motor (Ext Dig Brev) 28.9 C	Ankle	NR [<6.6]	NR [>2.0]	NR	NR	NR	Fib head	Ankle	NR	0	NR [>41]
	Fib Head	NR [<6.6]	NR [>2.0]	NR	NR	NR					
	Pop Fossa	NR [<6.6]	NR [>2.0]	NR	NR	NR	Pop Fossa	Fib Head	NR	0	NR [>41]
L Peroneal TA Motor (Tib Ant) 28.8 C	B Fib Head	6.3 [<4.2]	5.4 [>5.1]	6.1	12.42	31.76	Poplit	B Fib Head	5.3	7.5	14 [>41]
	Poplit	11.6 [<6.8]	5.3 [>5.1]	6.1	113.83	31.91					
L Tibial Motor (Abd Hall Brev) 28.8 C	Ankle	9.4 [<6.1]	0.5 [>4.0]	0.5	12.03	1.79	Knee	Ankle	22.2	45	20 [>41]
	Knee	31.6 [<6.1]	0.3 [>4.0]	0.5	12.81	1.4					

**Table 2:** Electromyography. Abbreviations: Abd (Abductor), Poll Brev (Pollicis Brevis), Dig Min (Digiti Minimi), Ext Dig Brev (Extensor Digitorium Brevis), Tib Ant (Tibialis Anterior), Tib Post (Tibialis Posterior), Hall Brev (Hallicus Brevis), Dor Int (Dorsal Interosseous), Flex Car Rad (Flexor Carpi Radialis), Med (Medialis), Musculocut (Musculocutaneous), Per (Peroneal).

Muscle	Nerve	Root	Fibrillation	Amplitude	Duration	Polyphasic Potentials	Recruitment
Abd Poll Brev	Median	C8-T1	Absent	Incr + 1	Incr + 1	30–60%	Reduced + 1
1st Dor Int	Ulnar	C8-T1	Absent	Incr + 1	Incr + 1	30–60%	Reduced + 1
Flex Car Rad	Median	C6-7	Absent	Normal	Normal	<15%	Normal
Biceps	Musculocut	C5-6	Absent	Normal	Normal	<15%	Normal
Triceps	Radial	C6-8	Absent	Normal	Normal	<15%	Normal
Ext Dig Brevis	Deep Branch Per	L5-S1	Absent	Poor activation			
Tib Ant	Deep Branch Per	L4-5	Absent	Incr + 1	Incr + 1	30–60%	Reduced + 1
Tib Post	Tibial	L5-S1	Absent	Poor activation			
Gastrocnemius	Rami	L5-S1	Absent	Incr + 1	Incr + 1	15–30%	Reduced + 1
Vastus Med	Femoral	L2-4	Absent	Normal	Normal	<15%	Normal

## Data Availability

No additional data were created.
